# Cyclin‐dependent kinases: Masters of the eukaryotic universe

**DOI:** 10.1002/wrna.1816

**Published:** 2023-09-17

**Authors:** Aleksandra J. Pluta, Cécilia Studniarek, Shona Murphy, Chris J. Norbury

**Affiliations:** ^1^ Sir William Dunn School of Pathology University of Oxford Oxford UK

**Keywords:** CDK, cell cycle, cyclin, kinase, transcription

## Abstract

A family of structurally related cyclin‐dependent protein kinases (CDKs) drives many aspects of eukaryotic cell function. Much of the literature in this area has considered individual members of this family to act primarily either as regulators of the cell cycle, the context in which CDKs were first discovered, or as regulators of transcription. Until recently, CDK7 was the only clear example of a CDK that functions in both processes. However, new data points to several “cell‐cycle” CDKs having important roles in transcription and some “transcriptional” CDKs having cell cycle‐related targets. For example, novel functions in transcription have been demonstrated for the archetypal cell cycle regulator CDK1. The increasing evidence of the overlap between these two CDK types suggests that they might play a critical role in coordinating the two processes. Here we review the canonical functions of cell‐cycle and transcriptional CDKs, and provide an update on how these kinases collaborate to perform important cellular functions. We also provide a brief overview of how dysregulation of CDKs contributes to carcinogenesis, and possible treatment avenues.

This article is categorized under:

RNA Interactions with Proteins and Other Molecules > RNA‐Protein Complexes
RNA Processing > 3′ End Processing
RNA Processing > Splicing Regulation/Alternative Splicing

## Graphical abstract

A family of structurally related cyclin‐dependent protein kinases (CDKs) drives many aspects of eukaryotic cell function. Much of the literature in this area has considered individual members of this family to act primarily either as regulators of the cell cycle or transcription, but there is increasing evidence of overlap.

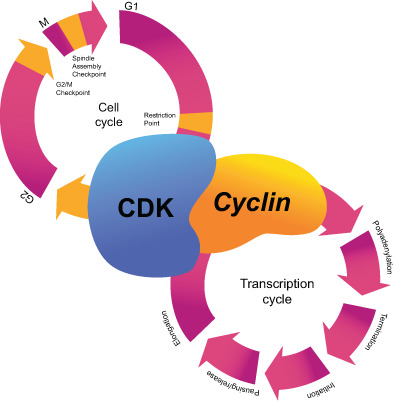

## 1 INTRODUCTION

**FIGURE 1:**
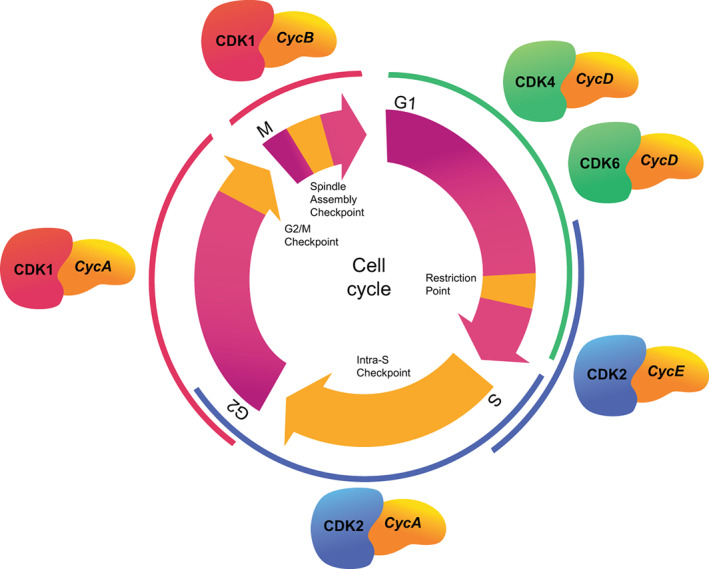
CDKs in the mammalian cell cycle. Different CDK‐cyclin complexes drive the individual phases of the cell cycle. Chromosomal DNA is replicated during the synthesis (S) phase; replicated chromosomes are segregated to daughter nuclei in M phase (mitosis). Gap phases G1 and G2 of variable duration may separate M from S and S from M, respectively. Mitogen‐dependent cells become committed to a new cycle and escape the requirement for mitogen signaling at the restriction (R) point in G1. In addition, progression through the cell cycle may be delayed by the presence of abnormal DNA structures at the point of DNA replication origin firing (the intra‐S checkpoint) or at the G2/M transition (the G2/M checkpoint), or by unattached chromosomes at the spindle attachment checkpoint in mitosis.

**FIGURE 2:**
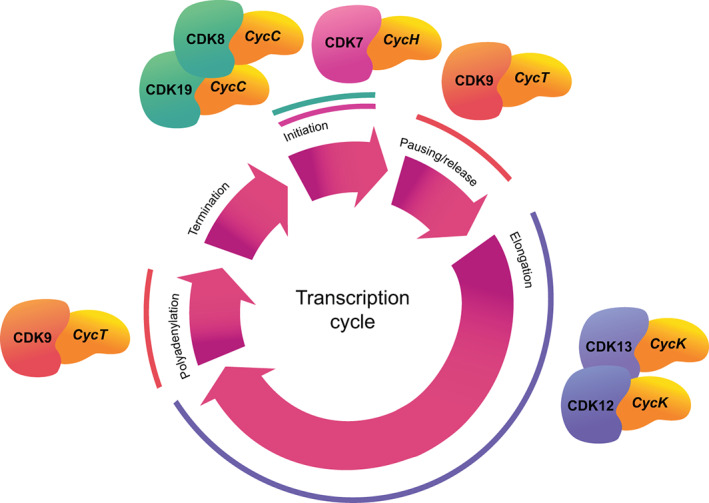
CDKs in transcription of mammalian protein‐coding genes. Individual CDK‐cyclin complexes are implicated in driving the sequential stages of transcription—initiation, pausing and release, elongation, polyadenylation and termination. The replication‐dependent histone genes have a specialized RNA 3′ processing signal rather than a poly(A) site but the cycle is thought to be similar (Marzluff & Koreski, 2017). The transcriptional CDKs influence transcription by phosphorylating the carboxy‐terminal domain of RNA polymerase II (pol II) and other members of the elongation complex. Additionally, CDK8/CDK19 form part of the mediator complex, which can promote or repress transcription.

**FIGURE 3:**
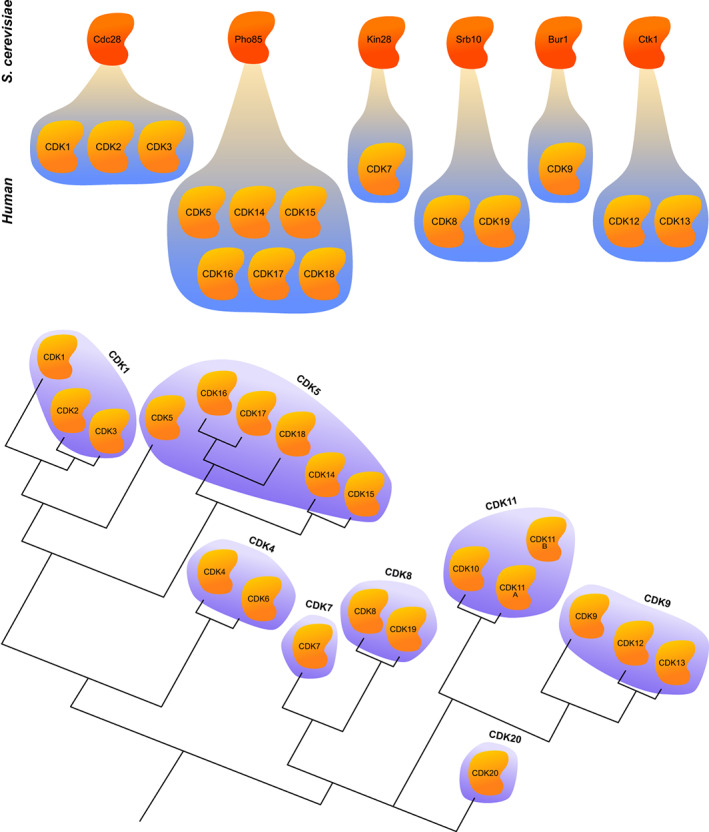
Evolution of CDKs. (a) *Saccharomyces cerevisiae* possesses the archetypal cell‐cycle CDK Cdc28, as well as Pho85, and four transcriptional CDKs—Kin28, Srb10, Bur1, and Ctk1. These kinases have 15 human CDK homologues. It is important to note that not all of the human CDKs have budding yeast homologues. (b) The relationships between human CDKs, based on their amino acid sequences, group them into eight subfamilies.

Cyclin‐dependent kinases (CDKs) are a conserved eukaryotic family of heterodimeric serine/threonine protein kinases, whose catalytic activity is entirely dependent on association with a specialized regulatory cyclin subunit and the phosphorylation status of the CDK activating domain, the T‐loop (Brown et al., 1999; Malumbres, 2014; Pines, 1995; Wood & Endicott, 2018). CDKs have been implicated in numerous processes in the cell, the most prominent being the cell cycle and the transcriptional cycle (Lim & Kaldis, 2013; Palmer & Kaldis, 2020) (Figures 1 and 2). These kinases function through dynamic and generally reversible phosphorylation of a wide range of targets, and 20 proteins belonging to this family (CDK1 to CDK20) have been identified in metazoans (Kalra et al., 2017; Malumbres et al., 2009; Marak et al., 2020; Roskoski, 2019) (Tables 1, 4, and 7). They can be further subdivided based on amino acid sequence and function into eight sub‐families (Cao et al., 2014; J. Liu & Kipreos, 2000; Malumbres, 2014; Figure 3).

**TABLE 1:** Cell‐cycle CDKs in metazoans.

Cyclin‐dependent kinase	Binding partner	Roles in cell cycle	Roles in transcription	Selected additional roles
CDK1	Cyclin A, B	Orchestrates mitosis and determines its timing (Maryu & Yang, 2022; Santamaría et al., 2007;Satyanarayana et al., 2008; Satyanarayana & Kaldis, 2009b)	Phosphorylates factors in cell‐cycle transcriptional programs (Akoulitchev & Reinberg, 1998; Chymkowitch & Enserink, 2013; Cisek & Corden, 1989; Enserink & Chymkowitch, 2022; Gavet & Pines, 2010a, 2010b; Kobor & Greenblatt, 2002) Maintains pluripotency of embryonic stem cells (mouse) (Michowski et al., 2020) Inhibits neuronal differentiation by phosphorylating Ngn2 (Ali et al., 2011) Phosphorylates of splicing and polyadenylation factors (in cell cycle‐dependent manner) (Colgan et al., 1998; Okamoto et al., 1998)	Regulates mitochondrial bioenergetics (B. Xie et al., 2019) Upregulates mRNA translation (Haneke et al., 2020) Functions in DNA damage response and DNA repair (Palmer & Kaldis, 2020)
CDK2	Cyclin A, E	Participates in G1/S transition (Satyanarayana et al., 2008; Satyanarayana & Kaldis, 2009b) Orchestrates S phase events, such as initiation of replication (Coverley et al., 2000)	Targets transcriptional repressor Rb (Dynlacht, 1997; Hydbring et al., 2016) Inhibits FOXO1 to promote cell survival (H. Huang et al., 2006) Activates ELK4 to stimulate cell transformation (Peng et al., 2016) Stimulates transcription during HIV‐1 infection (Agbottah et al., 2006; Deng et al., 2002; Nekhai et al., 2002; Rice, 2018) Targets chromatin‐modifying proteins and general transcription factors (e.g., DOT1L, GTF2I) (Chi et al., 2020) Inhibits neuronal differentiation by phosphorylating Ngn2 (Ali et al., 2011)	Maintains pluripotent neural progenitor cell pool (Caillava et al., 2011) Functions in DNA damage response and DNA repair (Satyanarayana & Kaldis, 2009a)
CDK4	Cyclin D	Governs progression through R‐point (Malumbres & Barbacid, 2005; Satyanarayana & Kaldis, 2009b)	Targets transcriptional repressors (Rb, p107, p130) and Smad transcription factors (Cobrinik, 2005; Dynlacht, 1997; Ezhevsky et al., 1997; Goel et al., 2018; Hydbring et al., 2016) Activates FOXM1 to protect against senescence (Anders et al., 2011) Activates c‐Jun to form AP‐1 transcription complexes (Vanden Bush & Bishop, 2011)	
CDK6	Cyclin D	Governs progression through R‐point (Malumbres & Barbacid, 2005; Satyanarayana & Kaldis, 2009b)	Targets transcriptional repressors (Rb, p107, p130) and Smad transcription factors (Cobrinik, 2005; Dynlacht, 1997; Ezhevsky et al., 1997; Goel et al., 2018; Hydbring et al., 2016) Activates FOXM1 to protect against senescence (Anders et al., 2011) Negatively regulates differentiation (Grossel & Hinds, 2006; Matushansky et al., 2000; Urbach & Witte, 2019) Activates NF‐κB (Buss et al., 2012; Handschick et al., 2014) Upregulates expression of pro‐angiogenic VEGF‐A (Kollmann et al., 2013)	Acts as sensitizer for apoptosis (MacKeigan et al., 2005)

**TABLE 2:** Cell‐cycle CDKs in *S. cerevisiae*.

Cyclin‐dependent kinase	Binding partner	Roles in cell cycle	Roles in transcription	Selected additional roles
Cdc28	Cln1‐3, Clb1‐6	Orchestrates mitosis and determines its timing; executes ‘START’ control in G1 and the initiation of DNA replication (Enserink & Kolodner, 2010; Hartwell et al., 1973; Mendenhall & Hodge, 1998)	Phosphorylates factors in cell‐cycle transcriptional programs (Archambault et al., 2004; R. J. Cho, Huang, et al., 2001; Cosma et al., 2001; Darieva et al., 2003; De Bruin et al., 2004; Enserink & Chymkowitch, 2022; Jans et al., 1995; Kõivomägi et al., 2011; Moll et al., 1991; O'Conalláin et al., 1999; Pic‐Taylor et al., 2004; Reynolds et al., 2003; Ubersax et al., 2003; Wittenberg & Reed, 2005) CTD kinase (T4, S5) (Chymkowitch & Enserink, 2013; Kõivomägi et al., 2021; Nemec et al., 2019) Recruits the proteasome to the promoter regions of certain genes (Morris et al., 2003; Yu et al., 2005). Maintains transcription of highly expressed housekeeping genes (e.g., PMA1) (Chymkowitch et al., 2012) Increases transcription through activating NuA4 leading to increased Lys14 acetylation on Htz1 (Fiedler et al., 2009) Recruits the proteasome to promoter regions of specific genes (Morris et al., 2003; V. P. C. C. Yu et al., 2005)	Implicated in bud morphogenesis (Enserink & Kolodner, 2010; Lew & Reed, 1993) Antagonizes pheromone signaling in G1 (Enserink & Kolodner, 2010) Governs genome stability and DNA repair pathways (Enserink & Kolodner, 2010)

CDKs in general are relatively long‐lived proteins and in most cases their levels do not markedly fluctuate through the cell cycle. However, the levels of their cyclin partners can vary over a wide range (Evans et al., 1983; Malumbres, 2014; Pines, 1995). Due to the crucial role of the CDK‐cyclin complexes in governing cell behavior, dysregulation of their activity often contributes to disease. As CDKs are involved in guarding both uncontrolled proliferation and genomic instability, they have become increasingly appealing as therapeutic targets in anticancer treatment (Malumbres & Barbacid, 2009).

## 2 CDKs DRIVE THE CELL CYCLE

The orderly replication and segregation of chromosomes during the cell cycle is governed by the sequential activation and inactivation of distinct CDK‐cyclin complexes. Three CDK‐dependent cell cycle transitions have been identified: the initiation of chromosomal DNA replication—in the S phase of the cell cycle (DNA synthesis), the initiation of mitosis—during M phase (usually followed by cell division), and cell cycle commitment in the G1 phase (Gap1), which in mammals is termed the restriction (R) point (Figure 1).

The orchestration of mitosis by CDK1 together with cyclin B is an essential aspect of eukaryotic cell cycle control conserved from fungi and plants to vertebrates. The universality of this function is underscored by the fact that human CDK1 was first identified through its capacity to complement loss of Cdc2 activity in fission yeast (Lee & Nurse, 1987). Interestingly, genetic inactivation of CDK1 in yeast and mammals not only blocks entry into mitosis but also de‐represses DNA replication, leading to endoreduplication of nuclear DNA (Hayles et al., 1994; Itzhaki et al., 1997). Germ‐line deletion of CDK1 or cyclin B in mice is associated with very early embryonic lethality (Santamaría et al., 2007), whereas tissue‐specific deletion of CDK1 in hepatocytes results in mitotic arrest, endoreduplication and cellular hypertrophy (Diril et al., 2012). In mammals, the essential role of CDK1 is supplemented by three “interphase” CDKs—CDK2, CDK4, CDK6 (Figure 1). These four kinases can bind to 10 individual cyclins, which in turn belong to four classes (A‐, B‐, D‐, and E‐cyclins) (Malumbres & Barbacid, 2009). Progression through the R point, beyond which cells have reduced dependency on mitogenic signaling, is attributed to activation of CDK4/6‐cyclin D complexes, while CDK2‐cyclin A/E activity is important for initiation of DNA replication (Malumbres, 2014). Each of the cell cycle CDKs is susceptible to inhibition by proteins of the Cip/Kip and/or Ink4 families, providing an additional level of regulation (Jeffrey et al., 2000; Kiyokawa & Koff, 1997; Lim & Kaldis, 2013; Pavletich, 1999; Sherr & Roberts, 1999). Further, all CDKs share two inhibitory phosphorylation sites in their ATP‐binding pocket, which are targeted by negative regulator kinases, and an activating T‐loop motif (Gould & Nurse, 1989; Lim & Kaldis, 2013; Loyer et al., 2005; Norbury et al., 1991).

Yeast CDK1 (originally named Cdc28 in budding yeast and Cdc2 in fission yeast) sequentially partners different cyclin proteins and can support all cell cycle functions (Nurse, 1990). Similarly, mouse development can proceed until mid‐gestation with CDK1 alone when CDKs 2, 4, and 6 are absent (Malumbres & Barbacid, 2009; Santamaría et al., 2007; Satyanarayana et al., 2008; Satyanarayana & Kaldis, 2009a, 2009b). The core cell cycle machinery is therefore conserved throughout eukaryotic evolution, despite the appearance of additional cell‐cycle CDKs in more recent evolutionary time (Tables 1, 2, 3).

**TABLE 3:** Cell‐cycle CDKs in *S. pombe*.

Cyclin‐dependent kinase	Binding partner	Roles in cell cycle	Roles in transcription	Selected additional roles
Cdc2	Cdc13, Cig1, Cig2, Puc1, Pas1, Pch1, Rem1	Governs cell cycle commitment at START in G1; orchestrates mitosis and determines its timing (Beach et al., 1982; Coudreuse & Nurse, 2010; Fisher & Nurse, 1996; Gutiérrez‐Escribano & Nurse, 2015; Martín‐Castellanos et al., 1996, 2000; Nurse, 1975; Nurse & Bissett, 1981; Reymond et al., 1993)	Required for Cdc10/Sct1 transcription complex formation (Connolly et al., 1997) Inhibits Ste11 (Kjærulff et al., 2007)	

## 3 TRANSCRIPTIONAL CDKs DRIVE THE TRANSCRIPTION CYCLE

In eukaryotes, transcription of protein‐coding genes by RNA polymerase II (pol II) can be characterized as a cycle of initiation, pausing, elongation, and termination (Figure 2). Each of these phases requires a different set of transcriptional machinery‐associated factors (Buratowski, 2009; Shandilya & Roberts, 2012; Svejstrup, 2004; Zaborowska et al., 2016). The best‐characterized transcriptional (t)CDKs to date are CDK7, CDK8, CDK9, CDK12, CDK13, and the CDK8 paralogue CDK19 (Table 4). These CDKs not only coordinate the transcription cycle, but also regulate co‐transcriptional processes, such as 5′ end capping, splicing, 3′ end cleavage and polyadenylation, termination, and regulation of the chromatin landscape (Chou et al., 2020; Fisher, 2017; Svejstrup, 2004; Zaborowska et al., 2016). One well‐studied target of the tCDKs is the carboxy‐terminal domain (CTD) of the largest pol II subunit, RPB1. In humans, the CTD comprises 52 heptad repeats with the consensus motif Tyr_1_Ser_2_Pro_3_Thr_4_Ser_5_Pro_6_Ser_7_, with the serine and threonine residues subject to dynamic and reversible phosphorylation by the tCDKs (Bartkowiak & Greenleaf, 2011; Galbraith et al., 2019; Zaborowska et al., 2016) to produce patterns of phosphorylation termed the CTD code (Buratowski, 2003). CTD phosphorylation plays a major role in the recruitment of transcription and RNA processing factors at the right point of the transcription cycle (Harlen & Churchman, 2017; Meinhart et al., 2005; Zaborowska et al., 2016). However, each tCDK has a range of other targets, which are in the process of being fully characterized (Fan et al., 2020; Krajewska et al., 2019; Larochelle et al., 2012; Rimel et al., 2020; Sansó et al., 2016; Tellier et al., 2020, 2022).

**TABLE 4:** Transcriptional CDKs in metazoans.

Cyclin‐dependent kinase	Binding partner	Roles in cell cycle	Roles in transcription	Selected additional roles
CDK7	Cyclin H	CAK activity (for CDK1/2/4/6) (Bisteau et al., 2013; Fisher, 2005, 2012; Larochelle et al., 1998, 2007; Merrick et al., 2008; Schachter & Fisher, 2013) Required for cell cycle progression (Ganuza et al., 2012; Larochelle et al., 1998, 2007; Olson et al., 2019; Wallenfang & Seydoux, 2002)	CAK activity (for CDK9/12/13) (Fisher, 2012; Larochelle et al., 2012; Rimel et al., 2020) Part of TFIIH complex, important for early steps of pol II transcription (Fisher, 2005; Glover‐Cutter et al., 2009; Larochelle et al., 2012; Maldonado & Reinberg, 1995; Rimel & Taatjes, 2018; Roy et al., 1994) CTD kinase (S5, S7) (Akhtar et al., 2009; Fisher, 2005; Glover‐Cutter et al., 2009; Pinhero et al., 2004; Roy et al., 1994; Wallenfang & Seydoux, 2002)	Implicated in DNA damage and repair pathways (Rimel & Taatjes, 2018)
CDK8	Cyclin C	Inhibits CAK activity (Akoulitchev et al., 2000; Szilagyi & Gustafsson, 2013) Promotes cell‐cycle commitment through β‐catenin pathway (Firestein et al., 2008; Szilagyi & Gustafsson, 2013) Promotes G2/M transition (Philip et al., 2018; Xu et al., 2015) Promotes cell cycle arrest at R point through p21 activation (Donner et al., 2007; Porter et al., 2012; Szilagyi & Gustafsson, 2013) Maintains quiescence of VPC in *C. elegans* (Clayton et al., 2008)	Part of mediator complex (Dannappel et al., 2019; Fant & Taatjes, 2019; Galbraith et al., 2010; Luyties & Taatjes, 2022; Poss et al., 2013; D. Wu, Zhang, et al., 2021) CTD kinase (S5) (Pinhero et al., 2004; Rickert et al., 1999) Can upregulate and downregulate transcription (Galbraith et al., 2010) Inhibits TFIIH to downregulate transcription (Akoulitchev et al., 2000) Promotes expression of hypoxia‐inducible genes after recruitment by HIF1A (Galbraith et al., 2013)	Acts as sensitiser for apoptosis (MacKeigan et al., 2005) Regulates Myc to maintain stem cell pluripotency (Adler et al., 2012) Essential for early embryogenesis (mouse) (Westerling et al., 2007) Implicated in DNA damage and repair pathways (Poss et al., 2016)
CDK19	Cyclin C		Part of mediator complex (Dannappel et al., 2019; Fant & Taatjes, 2019; Luyties & Taatjes, 2022)	Implicated in DNA damage and repair pathways (Poss et al., 2016)
CDK9	Cyclin T	Necessary for cell cycle recovery after replication stress (D. S. Yu et al., 2010) Upregulates cell proliferation through PABIR1 and PCNP (Tellier et al., 2022) Gets recruited to chromatin to promote progression through G1 (Anshabo et al., 2021; Cai et al., 2006; Storch & Cordes, 2016; Yang et al., 2008)	Part of P‐TEFb complex, important for pol II release from the promoter‐proximal pause and elongation (Anshabo et al., 2021; Bacon & D'Orso, 2019; Egloff, 2021; Jonkers & Lis, 2015; Luo et al., 2012; Parua et al., 2018) CTD kinase (S2, T4, S5, S7) (Eick & Geyer, 2013; Heidemann et al., 2013; Pinhero et al., 2004; Zaborowska et al., 2016) Targets transcriptional repressor Rb (Graña et al., 1994; Simone et al., 2002; Storch & Cordes, 2016)	Implicated in DNA damage and repair pathways (Anshabo et al., 2021)
CDK12	Cyclin K	Phosphorylates cyclin E1 to stimulate formation of pre‐replicative complex, therefore controlling G1/S progression (Lei et al., 2018; S. Liang et al., 2020; Manavalan et al., 2019) Controls translation of mitotic‐regulator gene mRNAs (Choi et al., 2019) Promotes G2/M transition (Blazek et al., 2011; Geng et al., 2019; H. R. Chen et al., 2017; Schecher et al., 2017) Downregulates G2/M transition in breast cancer cells (Quereda et al., 2019)	Required for efficient elongation and pre‐mRNA 3′ end formation (Bösken et al., 2014; Choi et al., 2020; Eifler et al., 2015; Fan et al., 2020; Lui et al., 2018; Quereda et al., 2019; Tellier et al., 2020) CTD kinase (S2, S5) (Bartkowiak et al., 2010; Blazek et al., 2011; Bösken et al., 2014; Cheng et al., 2012; Tellier et al., 2020) Implicated in regulation of splicing (H. H. Chen et al., 2006; K. Liang, Gao, et al., 2015; S. Liang et al., 2020; Panzeri et al., 2013) Inhibition severely affects expression of long DNA Damage Response (DDR) genes (Blazek et al., 2011; Choi et al., 2020; Dubbury et al., 2018; Krajewska et al., 2019) Negatively regulates differentiation (Dai et al., 2012)	Implicated in DNA damage response (Blazek et al., 2011; H. R. Chen et al., 2017; Choi et al., 2020; Dubbury et al., 2018; Fan et al., 2020; Juan et al., 2015; Krajewska et al., 2019; K. Liang, Gao, et al., 2015; S. Liang et al., 2020; Manavalan et al., 2019; Quereda et al., 2019; Tellier et al., 2020) Promotes neurogenesis (H. R. Chen et al., 2017) Essential for early embryogenesis (mouse) (Juan et al., 2015)
CDK13	Cyclin K	Promotes G2/M transition in gastric cancer cells (Z. Wu, Wang, et al., 2021) Downregulates G2/M transition in breast cancer cells (Quereda et al., 2019)	Required for efficient elongation and pre‐mRNA 3′ end formation (Fan et al., 2020; K. Liang, Gao, et al., 2015; Quereda et al., 2019) CTD kinase (S2, S5) (Greifenberg et al., 2016) Regulates expression of snoRNA genes (K. Liang, Gao, et al., 2015) Implicated in regulation of splicing (H. H. Chen et al., 2007; K. Liang, Gao, et al., 2015; Panzeri et al., 2013) Activates RNA surveillance pathway (Insco et al., 2023) Negatively regulates differentiation (Dai et al., 2012) Increases splicing of HIV‐1 mRNA (Berro et al., 2008)	Implicated in DNA damage response (Fan et al., 2020) Implicated in growth signaling pathways (Fan et al., 2020; Greifenberg et al., 2016) Promotes cell survival in breast cancer cells (Quereda et al., 2019) Promotes neurogenesis (H. R. Chen et al., 2017)

**TABLE 5:** Transcriptional CDKs in *S. cerevisiae*.

Cyclin‐dependent kinase	Binding partner	Roles in cell cycle	Roles in transcription	Selected additional roles
Kin28	Ccl1	Essential for proliferation (Simon et al., 1986) Binds to SBF promoters and can rescue cell cycle defects caused by Cln3 depletion (Kõivomägi et al., 2021) Creates autoregulatory loop with Srb10 to govern meiosis (Ohkuni & Yamashita, 2000)	Part of TFIIH complex, important for early steps of pol II transcription (Cismowski et al., 1995; Feaver et al., 1994; Svejstrup et al., 1996) Upregulates transcription (Hengartner et al., 1998; Valay et al., 1995) CTD kinase (S5, S7) (Akhtar et al., 2009; Hengartner et al., 1998; Komarnitsky et al., 2000) Important for recruitment of mRNA processing machinery (e.g., for 5′ capping) (Komarnitsky et al., 2000; Rodriguez et al., 2000; Schroeder et al., 2000) Primes CTD for Bur1 recruitment (Qiu et al., 2009, 2012) Stimulates mediator disassociation from preinitiation complex leading to promoter escape (Y. Liu, Wu, & Galaktionov, 2004; Wong et al., 2014)	Participates in nucleotide excision repair (Bhatia et al., 1996)
Srb10	Srb11	Represses meiotic genes in response to glucose by decreasing their mRNA stability (Surosky et al., 1994) Creates autoregulatory loop with Kin28 to govern meiosis (Ohkuni & Yamashita, 2000) Coordinates entry into stationary phase (Chang et al., 2001)	Part of mediator complex (Carlson, 1997) Downregulates transcription, e.g., through interaction with Gcn4 (Carlson, 1997; Chi et al., 2001; Hengartner et al., 1998; Holstege et al., 1998; Kuchin & Carlson, 1998; Rosonina et al., 2012) Upregulates transcription (Andrau et al., 2006; Galbraith et al., 2010; Y. Liu, Wu, & Galaktionov, 2004; X. Zhu et al., 2006) CTD kinase (S5) (Hengartner et al., 1998; Liao et al., 1995) Phosphorylates and decreases stability of Ste12 in normal nitrogen conditions (Nelson et al., 2003) Represses a‐specific genes in α cells (Wahi & Johnson, 1995) Activates Sip4 in nonfermentable carbon sources (Vincent et al., 2001)	Participates in glucose repression (Kuchin et al., 1995) Coordinates nutrient starvation response (Holstege et al., 1998)
Bur1	Bur2	Required for activation of Sch9 leading to progression through G1 (Jin et al., 2022)	Required for efficient elongation (Keogh et al., 2003; Murray et al., 2001; Wood et al., 2005; Wood & Shilatifard, 2006; Yao et al., 2000) CTD kinase (S2, S5, S7) (Y. Liu et al., 2009; Murray et al., 2001; Qiu et al., 2009; Tietjen et al., 2010) Phosphorylates Spt5 to recruit PAF1 (Y. Liu et al., 2009; Qiu et al., 2012) Represses SUC2 basal promoter (Prelich & Winston, 1993) Regulates epigenetic histone modifications, e.g., through PAF recruitment (Chu et al., 2006, 2007; Laribee et al., 2005; Y. Liu et al., 2009; Wood et al., 2005) Modulates co‐transcriptional splicing (Maudlin & Beggs, 2021)	Governs DNA damage and replication stress pathways (Clausing et al., 2010) Implicated in lengthening of telomeres (Connelly et al., 2022) Essential for cell growth (Irie et al., 1991; Winzeler et al., 1999) Suppresses mating pheromone hyperadaptivity (Irie et al., 1991)
Ctk1	Ctk2, Ctk3	Role unclear—deletion results in cell cycle defects (Chymkowitch et al., 2012)	Required for efficient elongation (Jona et al., 2001; J. M. Lee & Greenleaf, 1997; Murray et al., 2001) Regulates co‐transcriptional processes such as mRNA 3′‐end processing, polyadenylation, and nuclear export of mRNA (Ahn et al., 2004; Hurt et al., 2004; Skaar & Greenleaf, 2002) Promotes release of basal transcription factors from pol II (Ahn et al., 2009) CTD kinase (S2) (E. J. Cho, Kobor, et al., 2001; J. M. Lee & Greenleaf, 1989, 1991) Downregulates CTD phosphorylation during logarithmic phase growth, but upregulates during diauxic phase (Patturajan et al., 1999) Important for transcription termination of small non‐coding RNAs (Lenstra et al., 2013) Upregulates H3K36me3 by interaction with Set2 and Spt6 (Dronamraju & Strahl, 2014; Xiao et al., 2003; Youdell et al., 2008) Implicated in glucose‐dependent transcriptional regulation (van Driessche et al., 2005)	Implicated in cell growth (J. M. Lee & Greenleaf, 1991) Required for translation initiation and elongation (Coordes et al., 2015; Röther & Sträßer, 2007) Implicated in transcription by RNA pol I and synthesis of rRNA (Bouchoux et al., 2004; Grenetier et al., 2006) Implicated in DNA damage response (Ostapenko & Solomon, 2003)

In contrast to cell‐cycle CDKs, tCDKs usually have a single cyclin partner and are recruited to the transcriptional machinery as part of larger protein complexes (Galbraith et al., 2019) (Tables 4, 5, 6). For example, CDK7 associates with cyclin H and MAT1 to form the ternary kinase module of the TFIIH complex, a general transcription factor (TF) required for early steps of RNA pol II transcription (Glover‐Cutter et al., 2009; Maldonado & Reinberg, 1995; Rimel & Taatjes, 2018; Roy et al., 1994). CDK8, together with its paralogue CDK19, form the mediator complex kinase module through binding to cyclin C, MED12 and MED13 (Luyties & Taatjes, 2022). Together, this complex can regulate pol II activity through direct interaction with the transcriptional machinery, with activating or repressing functions depending on the context (Dannappel et al., 2019; Fant & Taatjes, 2019; Luyties & Taatjes, 2022; Parua & Fisher, 2020; D. Wu, Zhang, et al., 2021). Further, CDK9 and cyclin T make up positive transcription elongation factor b (P‐TEFb), which associates with a variety of transcription factors and coactivators, and forms part of the Super Elongation Complex (Bacon & D'Orso, 2019; Egloff, 2021; Luo et al., 2012). P‐TEFb kinase activity releases pol II from a promoter‐proximal pause into productive elongation by phosphorylating negative elongation factors associated with pol II (Jonkers & Lis, 2015; Parua et al., 2018; Parua & Fisher, 2020; Zaborowska et al., 2014). Finally, the most recent kinases described as bona fide tCDKs are CDK12 and CDK13. CDK12 is required for efficient elongation and pre‐mRNA 3′ end formation through recruitment of elongation and polyadenylation factors (Bösken et al., 2014; S. H. Choi et al., 2020; Greenleaf, 2019; Lui et al., 2018; Tellier et al., 2020). Although both bind to cyclin K and are redundant for some functions, including CTD phosphorylation and the regulation of the DNA damage response pathway (Fan et al., 2020; Greenleaf, 2019; Krajewska et al., 2019), the exact role(s) of CDK13 in transcription is less clear. As its individual role is being gradually characterized, it is becoming clearer that despite their structural similarities, the function of CDK13 is distinct from CDK12 and they can affect different sets of genes (Fan et al., 2020; Greifenberg et al., 2016; K. Liang, Gao, et al., 2015). However, both CDK12 and CDK13 have been shown to regulate splicing and to phosphorylate the CTD on Ser2 and Ser5 (H. H. Chen et al., 2007; H. H. Chen et al., 2006; Galbraith et al., 2019; Panzeri et al., 2013; Zaborowska et al., 2016). Recently, CDK13 was also found to play a critical role in the nuclear RNA surveillance pathway (Insco et al., 2023).

**TABLE 6:** Transcriptional CDKs in *S. pombe*.

Cyclin‐dependent kinase	Binding partner	Roles in cell cycle	Roles in transcription	Selected additional roles
Mc6	Mcs2	CAK activity (for Cdc2) (Buck et al., 1995; Damagnez et al., 1995; Lee et al., 1999; Saiz & Fisher, 2002) Inhibition impairs cytokinesis (Buck et al., 1995; Saiz & Fisher, 2002; Viladevall et al., 2009)	Part of TFIIH complex, important for early steps of pol II transcription (Booth et al., 2018; Spåhr et al., 2003; Viladevall et al., 2009) CTD kinase (S5, S7) (Amour et al., 2012; Booth et al., 2018; Viladevall et al., 2009) Recruits P‐TEFb to pol II by priming the CTD, leading to mRNA capping (Amour et al., 2012; Viladevall et al., 2009) Controls transcription of cell‐cycle periodic genes through interactions with Sep1 (Lee et al., 2005)	Essential for growth (Damagnez et al., 1995; Molz et al., 1989; Saiz & Fisher, 2002)
Srb10	Srb11	Controls entry into mitosis by phosphorylating Fkh2 (Banyai et al., 2014; Szilagyi et al., 2012)	Part of mediator complex (Borggrefe et al., 2002; Samuelsen et al., 2003; Spåhr et al., 2003) Downregulates transcription in vitro (Spåhr et al., 2003) CTD kinase in vitro (S2, S5) (Borggrefe et al., 2002) Blocks mediator‐pol II interactions (Elmlund et al., 2006; Samuelsen et al., 2003) Acts as a global regulator of mitotic transcription (Banyai et al., 2014)	Downregulates expression of adhesins (Linder et al., 2008; Samuelsen et al., 2003)
SpCDK9	Pch1	Inhibition impairs cytokinesis (Viladevall et al., 2009)	Part of P‐TEFb complex, important for efficient elongation (Amour et al., 2012; Bartkowiak & Greenleaf, 2011; Guiguen et al., 2007; Parua et al., 2018; Viladevall et al., 2009) Regulates an early elongation checkpoint (Booth et al., 2018; Guiguen et al., 2007; Pei et al., 2003; Viladevall et al., 2009) CTD kinase (S2, S5, marginally S7) (Amour et al., 2012; Guiguen et al., 2007; Pei & Shuman, 2003) Phosphorylates Spt5 elongation factor (Amour et al., 2012; Booth et al., 2018; Parua et al., 2018; Pei & Shuman, 2003) Couples transcription to mRNA capping through its association with Pct1 and Pcm1 (Amour et al., 2012; Guiguen et al., 2007; Pei et al., 2003, 2006; Viladevall et al., 2009) Downregulates PP1 isoform Dis2 to create a termination switch (Parua et al., 2018) Upregulates histone H2B mono‐ubiquitylation (Sansó et al., 2012)	Essential for growth (Bimbó et al., 2005; Guiguen et al., 2007; Pei & Shuman, 2003) Implicated in DNA damage response (Gerber et al., 2008)
Lsk1	Lsc1	Upregulates the Septation Initiation Network to promote cytokinesis (Karagiannis et al., 2005; Karagiannis & Balasubramanian, 2007) Upregulates meiosis via stress‐responsive MAPK pathway (Coudreuse et al., 2010; Sukegawa et al., 2011)	Deletion causes minimal changes in transcription (Booth et al., 2018) CTD kinase (S2) (Booth et al., 2018; Karagiannis & Balasubramanian, 2007; Viladevall et al., 2009)	Downregulates sexual differentiation through interaction with Ste11 (Coudreuse et al., 2010; Sukegawa et al., 2011)

## 4 OTHER ROLES FOR CDKs

CDKs are implicated in a variety of cellular processes in addition to the cell or transcription cycles. This is true both for metazoans and yeast, with CDK1 homologues alone implicated in cell morphogenesis and polarity, genome stability, telomere maintenance, and pheromone signaling in *Saccharomyces cerevisiae*, and mitochondrial bioenergetics, and positive regulation of mRNA translation in humans (Enserink & Kolodner, 2010; Haneke et al., 2020; Xie et al., 2019). CDKs have also been recently found to mediate inflammatory responses in mammals (Sundar et al., 2021). They also govern parts of the DNA damage response pathways, including DNA repair and damage checkpoint signaling (Hydbring et al., 2016; Palmer & Kaldis, 2020). Interestingly, CDKs may even perform functions independent of their kinase activity: CDK6 was demonstrated to upregulate the expression of p16INK4a and VEGF‐A, while the budding yeast CDK1 homologue—Cdc28—has been suggested to act as an adaptor protein during the recruitment of the proteasome (Kollmann et al., 2013; V. P. C. C. Yu et al., 2005).

A novel and intriguing area of research focuses on the roles of CDKs in stem cell biology (Jirawatnotai et al., 2020), which may also uncover new ways in which CDKs are implicated in cancer. For example, there are similarities between undifferentiated stem cells and malignant progenitor cells which are relatively undifferentiated but which give rise to more differentiated progeny within the tumor. CDK8 is implicated in cancer cell de‐differentiation and helps to maintain stem cell pluripotency due to regulation of the Myc proto‐oncogene (Adler et al., 2012; Peyressatre et al., 2015). Similarly, CDK2 seems to play a role in maintaining a pluripotent neural progenitor cell pool (Caillava et al., 2011; Chi et al., 2020). Relevant to this, CDK2 knockout mice are viable, but their neural progenitor cells display impaired proliferation in adults (Satyanarayana & Kaldis, 2009b).

## 5 LESS‐STUDIED CDKs

Additional CDKs have less well‐understood functions (Tables 7, 8, 9). However, it is worth noting that most of them have reported roles in both cell cycle and transcription (Chou et al., 2020; Kasten & Giordano, 2001; Lim & Kaldis, 2013; Loyer & Trembley, 2020; Malumbres & Barbacid, 2005, 2009; Trembley et al., 2003; Zheng et al., 2008) (Tables 7, 8, 9). For instance, apart from its involvement in transcription and splicing (Dickinson et al., 2002; Hluchý et al., 2022; Hu et al., 2003; Loyer & Trembley, 2020; Malumbres & Barbacid, 2009; Trembley et al., 2003), CDK11 is required for transcription of replication‐dependent histone genes during S phase, and depletion of this kinase induces accumulation of cells in G1 (Gajdušková et al., 2020). A separate CDK11p58 isoform functions at the G2/M transition (Hu et al., 2007; Petretti et al., 2006). The roles of these kinases emphasize that CDKs are versatile and not always easily pigeonholed.

**TABLE 7:** Additional CDKs in metazoans.

Cyclin‐dependent kinase	Binding partner	Roles in cell cycle	Roles in transcription	Selected additional roles
CDK3	Cyclin A, C, E	Promotes G0/G1 and G1/S transitions (Ren & Rollins, 2004; Sage, 2004; Satyanarayana & Kaldis, 2009b; Teo et al., 2022; van den Heuvel & Harlow, 1993; Ye et al., 2001)	Targets transcriptional repressor Rb (Hofmann & Livingston, 1996; Ren & Rollins, 2004) Phosphorylates c‐Jun and ATF1 to simulate cell transformation (Cho et al., 2009; Zheng et al., 2008)	Promotes apoptosis (Meikrantz & Schlegel, 1996) Promotes EMT (Lu et al., 2016)
CDK5	p35, p38	Blocks cell cycle in postmitotic neurons (Cicero & Herrup, 2005; Zhang et al., 2008) Promotes proliferation in neuroendocrine thyroid cancer (Pozo et al., 2013)	Implicated in transcriptional programs for neuronal differentiation (Cicero & Herrup, 2005) Activates STAT3 (A. K. Y. Fu et al., 2004) Inhibits MEF2 to promote apoptosis (Gong et al., 2003) Activates mSds3 to promote histone acetylation (Z. Li, David, et al., 2004) Activates p53 and Rb (Lee et al., 1997; Pozo et al., 2013; Zhang et al., 2002)	Implicated in a wide range of neuronal processes (Chae et al., 1997; Cicero & Herrup, 2005; Cruz & Tsai, 2004; Maestre et al., 2008; Nikolic et al., 1996; Ohshima et al., 1996; Pao & Tsai, 2021; Tanaka et al., 2001) Regulates circadian rhythm through phosphorylation of CLOCK and PER2 (Brenna et al., 2019; Kwak et al., 2013; Pao & Tsai, 2021) Protects against mitochondrial dysfunction and oxidative stress (Pao & Tsai, 2021; Qu et al., 2007; K. H. Sun et al., 2008) Implicated in DNA damage response (Kim et al., 2008; W. Liu, Li, et al., 2017)
CDK10	Cyclin M	Promotes G2/M transition (S. Li et al., 1995)	Phosphorylates ETS2, leading to silencing of the MAPK pathway (Guen et al., 2013; Kasten & Giordano, 2001) Promotes transcription of 20E‐inducible genes (W. Liu et al., 2014)	Regulates cilium biogenesis and degradation (Guen et al., 2016, 2018; Windpassinger et al., 2017) Regulates the cytoskeleton and actin dynamics (Guen et al., 2016, 2018)
CDK11	Cyclin L	Participates in G2/M transition and cytokinesis (primarily CDK11p58 isoform) (An et al., 2020; Barna et al., 2008; Hu et al., 2007; Loyer & Trembley, 2020; Petretti et al., 2006; Wilker et al., 2007; Yokoyama et al., 2008) Required for transcription of replication‐dependent histone genes during S phase (Gajdušková et al., 2020)	Associates with multiple transcription elongation factors (Loyer & Trembley, 2020; Trembley et al., 2002, 2003) Links transcription with RNA processing events (Loyer & Trembley, 2020; Trembley et al., 2002, 2003) Implicated in regulation of splicing (Hluchý et al., 2022; Hu et al., 2003; Loyer et al., 2005; Loyer & Trembley, 2020; Shin & Manley, 2004) CTD kinase in vitro (S2) for replication‐dependent histone genes (Gajdušková et al., 2020) Involved in processing and polyadenylation of HIV‐1 transcripts (Pak et al., 2015; Rice, 2018)	Part of autophagy machinery (Wilkinson et al., 2011) Represses estrogen and vitamin D receptor pathways (Chi et al., 2009; Wang et al., 2009) Promotes apoptosis (Ariza et al., 1999; Beyaert et al., 1997; Lahti et al., 1995; J. Shi et al., 2003, 2009; L. Shi et al., 1994; Tang et al., 1998) Essential for early embryogenesis (mouse) (T. Li, Inoue, et al., 2004)
CDK14	Cyclin B, Y	Stimulates Wnt/β‐catenin signaling pathway to drive cell cycle progression (Gu et al., 2015; Niehrs & Acebron, 2012; Ou‐Yang et al., 2017; T. Sun et al., 2014)	Upregulates phosphorylation of Rb to deactivate it (L. Chen et al., 2019)	Implicated in tumor cell migration and EMT (L. Chen et al., 2019; Gu et al., 2015; Ou‐Yang et al., 2017; Pang et al., 2007; Zhu et al., 2016) Promotes axon regeneration (Hisamoto et al., 2021) Implicated in glucose homeostasis (Tang et al., 2006)
CDK15	Cyclin Y	Promotes cell proliferation through β‐catenin/MEK–ERK pathway in colorectal cancer (C. Huang et al., 2022)		Protects against tumor cell migration (S. Li, Dai, et al., 2019) Downregulates apoptosis (Park et al., 2014)
CDK16	Cyclin Y, p35	Promotes cell proliferation (Gillani et al., 2022; X. Li et al., 2022; Yanagi et al., 2014)	Deactivates p27 and p53 tumor suppressors (J. Xie et al., 2018; Yanagi et al., 2014)	Involved in terminal differentiation in spermatogenesis (Mikolcevic, Rainer, & Geley, 2012; Mikolcevic, Sigl, et al., 2012) Implicated in neural outgrowth (Graeser et al., 2002; Mokalled et al., 2010) Functions in vesicular trafficking (Y. Liu et al., 2006; Ou et al., 2010; Palmer et al., 2005; Shehata et al., 2019) Implicated in brain development (Cole, 2009; Fu et al., 2011; Le Bouffant et al., 2000; Mokalled et al., 2010; Shehata et al., 2015, 2019) Suppresses apoptosis (Gillani et al., 2022; Yanagi & Matsuzawa, 2015) Implicated in glucose homeostasis (X. Y. Chen et al., 2012; Tang et al., 2006) Promotes myogenesis (Shimizu et al., 2014)
CDK17	Cyclin Y		Phosphorylates Histone 1 (Hirose et al., 1997)	Promotes Alzheimer pathology (Chaput et al., 2016) Implicated in neuronal development (Hirose et al., 2000; Yamochi et al., 2001) Downregulates autophagy (Leonardi et al., 2019)
CDK18	Cyclin A	Induces cell cycle arrest in glioblastoma cells (Naumann et al., 2005) Required for S phase progression (Barone et al., 2016)		Promotes Alzheimer pathology, promotes phosphorylation of tau (Chaput et al., 2016; Herskovits & Davies, 2006) Downregulates autophagy (Leonardi et al., 2019) Promotes ATR‐dependent homologous recombination (Ning et al., 2019) Prevents accumulation of DNA damage and genome instability (Barone et al., 2016, 2018) Involved in reorganization of the actin cytoskeleton (Matsuda et al., 2014)
CDK20	Cyclin H	Possible CAK activity (for CDK2) (X. An et al., 2010; Y. Liu, Kung, et al., 2004; Tian et al., 2012; questioned by Wohlbold et al., 2006; Wu et al., 2009) Promotes G1/S transition, and to a lesser extent G2/M (Mok et al., 2018) Promotes progression through the cell cycle by cyclin D upregulation (Wu et al., 2009) and cyclin E (X. An et al., 2010) Stimulates Wnt/β‐catenin/TCF signaling pathway to drive cell cycle progression (Feng et al., 2011)	Upregulates EZH2 and H3K27me3 (Feng et al., 2015)	Phosphorylates MAK‐related kinase/intestinal cell kinase (MRK/ICK) to suppress apoptosis (Z. Fu et al., 2023) Regulates ciliogenesis, and Hedgehog signaling downstream (Snouffer et al., 2017; Y. Yang et al., 2013) Promotes cell growth and survival in different contexts (Lai et al., 2020) Promotes the establishment of an immunosuppressive tumor microenvironment (Mok et al., 2018; Zhou et al., 2018)

**TABLE 8:** Additional CDKs in *S. cerevisiae*.

Cyclin‐dependent kinase	Binding partner	Roles in cell cycle	Roles in transcription	Selected additional roles
Pho85	Pcl1, Pcl2, Pho80, Clg1, Pcl5–10	Required for G1 progression and cell cycle commitment in the absence of Cln1 and Cln2 (Espinoza et al., 1994; Measday et al., 1994, 1997) Phosphorylates Sic1 leading to its degradation and G1/S transition (Nishizawa et al., 1998) Phosphorylates Rim15 to prevent cells from entering G0 (Huang et al., 2007) Establishes mitotic spindle in M and interacts with spindle assembly checkpoint genes MAD1 and BUB3 (Daniel et al., 2006)	Deactivates Pho4 in high phosphate conditions (Huang et al., 2007; Lenburg & O'Shea, 1996) Phosphorylates Gcn4p to target it for degradation in high amino acid conditions (Carroll & O'Shea, 2002; Huang et al., 2007; Meimoun et al., 2000; Shemer et al., 2002) Phosphorylates Rim101 for export from the nucleus to regulate alkali stress response (Nishizawa et al., 2010) Phosphorylates Crz1 in high calcium conditions (Huang et al., 2007; Sopko et al., 2006)	Downregulates Gsy2 glycogen synthase (Carroll & O'Shea, 2002; Huang et al., 1998, 2007) Implicated in bud morphogenesis (Carroll & O'Shea, 2002; Huang et al., 2007; Moffat & Andrews, 2004) Downregulates autophagy (Wang et al., 2001) Regulates the actin cytoskeleton (J. Lee et al., 1998)

**TABLE 9:** Additional CDKs in *S. pombe.*

Cyclin‐dependent kinase	Binding partner	Roles in cell cycle	Roles in transcription	Selected additional roles
Pef1	Pas1, Clg1, Psl2	Promotes pre‐meiotic DNA replication (Matsuda et al., 2021) Upregulates cohesin binding during the cell cycle (Birot et al., 2020)	Activates Res2p‐Cdc10p on MCB genes (Tanaka & Okayama, 2000)	Regulates sexual differentiation through upregulation of Ste11 and balancing the TORC1 pathway and autophagy (Matsuda et al., 2020) Downregulates cellular lifespan (Chen et al., 2013)
CDK11	Lcp1		Upregulates assembly of the mediator complex (Drogat et al., 2012)	

## 6 STRUCTURE OF CDKs AND CYCLINS

Throughout evolution, the CDK family has maintained some important features among its members. A protein kinase can be classified as a CDK based on its structural similarities with the canonical CDKs, including the presence of a PSTAIRE‐like cyclin‐binding element in the catalytic domain (Lim & Kaldis, 2013; Loyer & Trembley, 2020; Malumbres, 2014; Malumbres et al., 2009; Pines, 1995). Cyclins, by comparison, share more limited amino acid sequence similarity (Wood & Endicott, 2018). They were first identified by and named after their marked cell cycle‐dependent degradation on exit from mitosis and subsequent resynthesis (Evans et al., 1983). It is now clear that this cyclical behavior is a feature of a minority of cyclins; members of the family are more generally identified through one or two repeats of a “cyclin box” motif which assumes a fold comprising five alpha helices (Pines, 1995; Wood & Endicott, 2018).

The structural characterization of the CDK family began with studies on the CDK2‐cyclin A complex in various activational states and was used as an overarching model for CDK activation and regulation (Echalier et al., 2010; Wood & Endicott, 2018). It has now become apparent that the CDK‐cyclin complexes are highly variable in their 3D organization, and that this original standard is not applicable to many of the family members (Peissert et al., 2020). In addition, the size of CDKs ranges from 297 amino acid residues in CDK1 to 1512 residues in the case of CDK13 (Kohoutek & Blazek, 2012; Malumbres, 2014; Malumbres & Barbacid, 2005; Marqués et al., 2000).

## 7 CELL CYCLE OR TRANSCRIPTION: A FALSE CDK DICHOTOMY

### 7.1 Half a century of CDKs

The archetypal CDKs were discovered in the 1970s and 1980s, through genetic analysis of the yeast cell cycle (Hartwell et al., 1973; Morgan, 1995; Nurse et al., 1976), while cyclins were first identified through investigations of cell cycle‐regulated protein synthesis in sea urchin embryos (Evans et al., 1983). With the realization that the mitosis‐promoting activity of CDK1 depends on its association with cyclins, the term “cyclin‐dependent kinase” was first coined in 1991, at which point it was widely assumed that other enzymes of this class might also be involved in cell cycle regulation (Draetta et al., 1989; Labbe et al., 1989; Malumbres & Barbacid, 2005; Nurse, 1990). With the ever‐increasing availability of cDNA and ultimately whole‐genome sequences, it quickly became apparent that there is a multiplicity of CDKs, even in single‐celled eukaryotes. Budding yeast have 6 CDKs and 23 cyclins, while mammals have at least 20, along with a complement of ~30 distinct cyclins (Cao et al., 2014; Ercan et al., 2021; D. Huang et al., 2007; King et al., 1996; J. Liu & Kipreos, 2000; Malumbres, 2014; Tables 1, 2, 3, 4, 5, 6, 7, 8, 9).

Parallel but independent lines of investigation led to the identification of CDK‐cyclin complexes involved in transcriptional regulation, notably CDK7‐cyclin H in TFIIH (Akoulitchev et al., 1995; Drapkin & Reinberg, 1994; Shiekhattar et al., 1995) and CDK9‐cyclin in T/P‐TEFb (Graña et al., 1994; Marshall et al., 1996; J. Peng et al., 1998; Peterlin & Price, 2006). These distinct routes to the identification of cell‐cycle CDKs on the one hand and tCDKs on the other inevitably suggested a binary distinction between CDKs based on these broad biological functions, but it is becoming increasingly clear that such a classification is overly simplistic, as discussed below.

### 7.2 Regulation of transcription through the cell cycle

During the cell cycle, specific transcriptional programs ensure its directionality and proper timing, creating an oscillating pattern of gene expression (Gottesfeld & Forbes, 1997; Johnson & Holland, 1965; Ramos‐Alonso et al., 2023; Segil et al., 1996; Zaret, 2014). A significant change occurs at the onset of M phase, when transcription is largely, although not completely, silenced. This mitotic repression of all RNA polymerases is a well‐documented phenomenon (Gottesfeld & Forbes, 1997; Johnson & Holland, 1965; Parsons & Spencer, 1997). The main driving force behind mitotic silencing is the loss of chromatin accessibility, which is achieved through chromatin condensation during prophase, and/or through a range of histone modifications (Gottesfeld & Forbes, 1997; Ramos‐Alonso et al., 2023). The second essential feature of transcriptional repression during mitosis is the displacement of transcription factors from chromatin, including even the actively engaged pol II (Gottesfeld & Forbes, 1997; K. Liang, Woodfin, et al., 2015; Timmers & Verrijzer, 2017).

Mitotic silencing requires the hyperphosphorylation of pol II, and release of the general factors TFIID and TFIIH from core promoters (Loyer et al., 2005; Loyer & Trembley, 2020; Segil et al., 1996). Thus, the phosphorylation status of pol II not only changes through the transcriptional cycle, but with the cell cycle as well. However, a low‐level transcriptional program remains active during mitosis, keeping a subset of genes ready for re‐entry into interphase (Palozola et al., 2017; Timmers & Verrijzer, 2017; Zaret, 2014). Thus, some general transcription factors, including TFIID, can be retained on some genes, in a process known as mitotic “bookmarking” (Y. Liu, Pelham‐Webb, et al., 2017; Teves et al., 2016). General transcription is subsequently re‐established in telophase (Gottesfeld & Forbes, 1997). With the clear need for communication between the cell and transcription cycles to ensure they occur in the right order, the emerging evidence of interconnected functions of the cell‐cycle and tCDKs could point to their key role in coupling these processes.

### 7.3 Cell‐cycle CDKs with direct transcriptional roles—CDK1 as a tCDK?

Progression through the consecutive stages of the cell cycle requires activation of distinct transcriptional programs; factors expressed at a particular stage of the cycle will activate processes and proteins important for the next phase, thus ensuring unidirectionality. The “cell‐cycle” CDKs play therefore important roles in transcriptional control. For example, in yeast the archetypal cell‐cycle kinase CDK1 activates transcriptional programs required for maintaining the directionality of the cell cycle (Chymkowitch et al., 2012; Cosma et al., 2001; Enserink & Kolodner, 2010). Depending on the cell‐cycle stage, budding yeast Cdc28 communicates with distinct complexes and proteins to perform its function. Although the direct transcriptional effects of Cdc28 still need to be fully characterized, it is known that, for instance, in G2 Cdc28 stimulates the expression of CLB2 cluster of genes through phosphorylation of forkhead transcription factor Fkh2 and rate‐limiting transcriptional transactivator Ndd1 (Cho, Huang, et al., 2001; Darieva et al., 2003; Pic‐Taylor et al., 2004; Reynolds et al., 2003; Wittenberg & Reed, 2005), while in M phase it regulates the MCM, SIC1, and MAT gene clusters by phosphorylating transcription factors such as Swi5, and Ace2 (Archambault et al., 2004; Cho, Kobor, et al., 2001; Jans et al., 1995; Moll et al., 1991; O'Conalláin et al., 1999). However, the best characterized impact of Cdc28 on transcription is during G1; here Cdc28 acts on genes bound by the transcription factors Mlu1‐box binding factor (MBF) and Swi4/6‐dependent box‐binding factor (SBF), which encode proteins involved in DNA repair and cell cycle progression (Enserink & Kolodner, 2010). Cdc28 recruits pol II, TFIIB, and TFIIH to promoter regions of these genes through phosphorylation of Whi5, which then dissociates from SBF leading to activation of genes responsible for cell cycle entry (Cosma et al., 2001; De Bruin et al., 2004; Enserink & Kolodner, 2010). Notably, Cdc28 is able to directly phosphorylate the pol II CTD at S5 at the SBF target genes, through a positive feedback loop with Kin28 (TFIIH; Enserink & Chymkowitch, 2022; Kõivomägi et al., 2021; Ubersax et al., 2003). However, in mutant yeast cells which do not express S phase or mitotic cyclins, almost 70% of cell cycle‐regulated genes are still activated on time, suggesting an independent transcriptional oscillator functions alongside Cdc28 (Haase & Reed, 1999; Orlando et al., 2008; Simmons Kovacs et al., 2008).

Apart from regulation of the cell cycle‐dictated transcriptional programs, there is growing evidence of the involvement of Cdc28 in governing basal transcription. In *S. cerevisiae*, Cdc28 activity regulates a number of highly expressed housekeeping genes, such as *PMA1* (Chymkowitch et al., 2012; Enserink & Chymkowitch, 2022; Serrano et al., 1982) by phosphorylating the S5 residue of pol II to upregulate transcription and promote the recruitment of capping factors. Again, it is able to perform this role through a mutual priming system with Kin28 (Chymkowitch et al., 2012; Cosma et al., 2001; Enserink & Chymkowitch, 2022; Kõivomägi et al., 2011). Cdc28 may phosphorylate the CTD not only at the S5 residue, but also at T4 in vivo and S2 in vitro (Nemec et al., 2019). Additionally, Cdc28 can regulate transcriptional processes in a more indirect manner; for instance, it phosphorylates the NuA4 chromatin modifier leading to increased Lys14 acetylation on histone Htz1 and increased transcription (Enserink & Kolodner, 2010; Fiedler et al., 2009).

Intriguingly, there appears to be a marked difference in the effect of Cdc28 on the transcriptional machinery in budding yeast and the effect of CDK1 on transcription in metazoans. In *S. cerevisiae*, the onset of M‐phase does not induce a dramatic shutdown of transcription; this may reflect the limited extent of mitotic chromatin condensation in this organism, which is also unusual by comparison with many other eukaryotes in that the key mitotic event of microtubular spindle assembly overlaps with S phase. Cdc28 usually serves to upregulate transcription, while CDK1 has a seemingly opposite function in human cells. The reasons behind this are not yet fully understood, but a possible explanation is that so far CDK1 studies were focused mainly on the transcriptional silencing that occurs during the M phase of the mammalian cell cycle, while the transcriptional actions of CDK1 in interphase have been largely overlooked (Enserink & Chymkowitch, 2022). However, it is important to note that the evolutionary ancestor of Cdc28 evolved into CDK1, CDK2, and CDK3 in metazoans, which complicates any direct functional comparison of Cdc28 and CDK1 (Figure 3).

It is clear that in human cells, as in yeast, CDK1 engages primarily in the control of cell‐cycle transcriptional programs to regulate cell division. For instance, during interphase, the CDK1‐cyclin A complex phosphorylates E2F to induce transcription of S‐phase genes, and its inhibition leads to E2F transcription factor‐dependent cell death (Shapiro, 2006). Although the CDK1‐cyclin B complex is thought to be mostly cytoplasmic during interphase, effects on transcription may be carried out by CDK1 partnered with a different cyclin, or simply indirectly (Gavet & Pines, 2010a, 2010b; Maryu & Yang, 2022). However, the best characterized transcriptional role of CDK1 is that of transcriptional silencing during M phase of the metazoan cell cycle. Unlike in budding yeast, where mutual phosphorylation between Cdc28 and Kin28 upregulates transcription, in human cells, CDK1 inhibits CDK7 through phosphorylation of the Ser164 residue in its T‐loop, therefore suppressing TFIIH‐dependent phosphorylation of the CTD (Akoulitchev & Reinberg, 1998; Cisek & Corden, 1989; Guo & Stiller, 2004; Kobor & Greenblatt, 2002; Long et al., 1998; Loyer & Trembley, 2020). Other direct mitotic phosphorylation targets of CDK1 include several general transcription factors, including TBP and TBP‐associated factors (Enserink & Chymkowitch, 2022; Long et al., 1998). CDK1 has also been shown to phosphorylate the pol II CTD in vitro (Enserink & Chymkowitch, 2022; Gebara et al., 1997; Xu et al., 2003; Xu & Manley, 2004; Zhang & Corden, 1991). However, it is not yet clear whether CDK1 phosphorylates the pol II CTD in vivo. It is worth mentioning that the first‐reported classical biochemical purification of a CTD kinase identified CDK1 (Chymkowitch & Enserink, 2013; Cisek & Corden, 1989; Pines, 1995).

Intriguingly, an unexpectedly high level of CDK1‐cyclin A activity in mouse embryonic stem cells phosphorylates multiple chromatin‐associated proteins to maintain the stem cell epigenetic landscape, and its inhibition leads to stem cell differentiation (Michowski et al., 2020). It remains to be seen if hyperactive CDK1 is similarly involved in the maintenance of cancer stem cell populations, but this discovery clearly underlines that cell‐cycle CDKs can regulate functions previously attributed purely to tCDKs (Sánchez‐Martínez et al., 2019). It also serves as further example of the involvement of CDKs in stem cell biology (see Section 4). In prostate cancer, ABCC5‐bound CDK1 has been found to directly phosphorylate the AR transcription factor to stimulate its activity, further highlighting that CDK1 might possess non‐mitotic transcriptional roles in different cellular contexts (Ji et al., 2021). CDK1 may also carry out cell cycle‐dependent phosphorylation of splicing factors and the polyadenylation machinery (Colgan et al., 1998; Okamoto et al., 1998). It is even conceivable that the key role of ancestral CDK1 lay in transcriptional regulation, and that functions of CDK1 in the more direct regulation of the mitotic apparatus evolved more recently (Chymkowitch & Enserink, 2013; Enserink & Chymkowitch, 2022).

### 7.4 The relationship of interphase cell‐cycle CDKs to transcription

The transcriptional roles of interphase CDKs in metazoans are also becoming increasingly evident. Progression through the R point requires CDK4/6 to phosphorylate the retinoblastoma protein (Rb), which is a broad‐specificity transcriptional repressor, and Rb‐related pocket proteins p107 and p130 (Dynlacht, 1997). In addition, substrates of CDK4/6 include other transcription factors, such as Smads (Anders et al., 2011; Cobrinik, 2005; Goel et al., 2018; Hydbring et al., 2016; Malumbres & Barbacid, 2001). CDK4/6‐dependent phosphorylation maintains activity of the Forkhead Box M1 (FOXM1) transcription factor, thereby preventing cells from entering senescence (Anders et al., 2011). CDK4 was further found to phosphorylate c‐Jun to form active AP‐1 transcription complexes in non‐dividing immune cells (Vanden Bush & Bishop, 2011). CDK6 is also implicated in influencing transcription during angiogenesis by upregulating expression of pro‐angiogenic VEGF‐A; and in pro‐inflammatory signaling where it phosphorylates and so activates the p65 subunit of NF‐κB (Handschick et al., 2014; Kollmann et al., 2013). Even further, CDK6 was found to phosphorylate and inactivate transcription factors which drive cell differentiation, for example in osteoblast and osteoclast cells or during neurogenesis (Grossel & Hinds, 2006; Urbach & Witte, 2019).

The CDK2‐cyclin E complex has several transcriptional targets including Rb and the transcription factor ELK4. Activation of ELK4 through phosphorylation leads to an increase in c‐fos expression, which facilitates malignant transformation in, for example, melanoma development (C. Peng et al., 2016). CDK2 also promotes cell survival in response to DNA damage through phosphorylation of the pro‐apoptotic TF FOXO1, which then relocates to the cytoplasm (H. Huang et al., 2006). Intriguingly, it has been found that CDK2 is recruited during interphase to stimulate transcription during human immunodeficiency virus 1 (HIV‐1) infection (Agbottah et al., 2006; Nekhai et al., 2002; Rice, 2018). To sustain HIV‐1 transcriptional elongation, CDK2 binds and phosphorylates the viral transactivator protein Tat, which in turn activates CDK7 leading to pol II clearance of the HIV‐1 proviral promoter. Tat also stimulates the recruitment of CDK9, and together these kinases phosphorylate the pol II CTD, which allows for the cell cycle‐dependent expression of HIV‐1 (Deng et al., 2002; Nekhai et al., 2002). CDK2 may also be involved in phosphorylation of Hepadnavirus core protein C‐terminal domain during human hepatitis B infection (Ludgate et al., 2012). Although CDK2 can phosphorylate the pol II CTD in vitro, it is unclear if it phosphorylates the pol II CTD in vivo outside of HIV‐1 infection (Guo & Stiller, 2004; Malumbres, 2014; Palancade & Bensaude, 2003). A recent study used the analogue‐sensitive kinase (AS) approach, which utilizes bulky ATP analogues carrying a transferable thiophosphate (Larochelle et al., 2006, 2007; Schachter & Fisher, 2013), to identify nuclear targets of CDK2 (Chi et al., 2020). Notably, several identified substrates were chromatin‐modifying proteins, such as the histone demethylase LSD1 and the histone methyltransferase DOT1L, in addition to several transcription factors, like the general TF GTF2I, or BCL11A and AF9, which are linked to cancer. This study implicates CDK2 directly in transcription regulation, which may provide an additional link to cell cycle control.

### 7.5 tCDKs with cell cycle roles

As the cell‐cycle CDKs clearly have a profound influence on transcription, it is logical to consider the reciprocal effect of tCDKs on the cell cycle. A good example of a CDK with dual roles is CDK7 (Table 4), which despite being traditionally classified as a tCDK, was initially identified as the metazoan CDK‐activating kinase (CAK), required for activation of both CDK1 and CDK2 by phosphorylating the “T‐loop” region in a way that is essential for their activity (Fisher, 2005). More recently, CDK7 was also shown to activate CDK4/6 (Schachter & Fisher, 2013) and tCDKs CDK9, 12 and 13 (Fisher, 2012; Larochelle et al., 2012; Rimel et al., 2020). Interestingly, when its central role in transcriptional regulation was first uncovered, its status as a CAK was questioned (Fisher, 2005; Harper et al., 1998). This may be due to the fact that its budding yeast homologue, Kin28, does not perform this function (Fisher, 2005, 2019; Malumbres, 2014). Not only do *S. cerevisiae* cells possess a specialized CAK responsible for activating CDK1 (and Kin28), but also they have a separate CAK which activates the tCDKs including the CDK9 homologue Bur1 (Espinoza et al., 1998; Fisher, 2019; Ostapenko & Solomon, 2005; Yao & Prelich, 2002). In multicellular organisms, however, CDK7 is indispensable for cell proliferation and development. For example, CDK7 activity is required for cell division in *Drosophila* and *C. elegans* (Larochelle et al., 1998; Wallenfang & Seydoux, 2002). In addition, CDK7 deficiency in mice leads to early‐embryonic lethality and premature aging of adult tissues with high proliferative ability, such as skin or intestinal epithelium (Ganuza et al., 2012). In human cells expressing the AS version of CDK7, CDK7 was shown to be essential for G1 phase progression, DNA replication and mitotic entry through activation of CDK4/6, CDK2, and CDK1, respectively (Bisteau et al., 2013; Larochelle et al., 2007; Schachter & Fisher, 2013). Similarly, inhibition of CDK7 with the selective inhibitor YKL‐5‐124 increased the number of cells in G1 and G2, with a concomitant decrease of cells in S phase (Olson et al., 2019). While CDK7 is considered constitutively active and stably expressed during the cell cycle, several mechanisms explain the timely and specific activation of the cell‐cycle CDKs. Firstly, mitogenic signals can trigger CDK7 T‐loop phosphorylation leading to an increase activity toward CDK4, which in turn phosphorylates Rb to promote R point transition (Schachter & Fisher, 2013). In addition, CDK7 has a preference for cyclin‐associated CDK1 but for the monomer of CDK2, which is likely to mediate the sequential activation of CDK2 followed by CDK1 (Larochelle et al., 2007; Merrick et al., 2008, 2011). Other factors, such as post‐translational modifications of cyclin H or MAT1, as well as association with its partners in TFIIH, are likely to account for CDK7 substrate specificity during the cell cycle (Akoulitchev et al., 2000; Rimel et al., 2020; Schneider et al., 2002).

Further, CDK8‐cyclin C can inhibit CAK activity through phosphorylation of cyclin H in vitro (Malumbres & Barbacid, 2005; Szilagyi & Gustafsson, 2013). CDK8 also targets p21, a broad‐specificity CDK inhibitor protein that represses cell cycle progression following exposure to a variety of stresses, including activation of the p53 tumor suppressor (Donner et al., 2007; Szilagyi & Gustafsson, 2013). CDK8 acts as a co‐activator of *WAF1*, the gene encoding p21, promoting cell‐cycle arrest at the R point and p21, in turn, directly stimulates the activity of CDK8, creating a potential positive feedback loop (Porter et al., 2012). Conversely, as a part of the mediator complex, CDK8 activates the β‐catenin transcriptional program, which promotes cell cycle commitment (Firestein et al., 2008; Szilagyi & Gustafsson, 2013). Although there is no evidence that CDK8/19 can influence the G2/M transition in metazoans, the CDK8 homologue in fission yeast phosphorylates Fkh2, which controls a cluster of genes expressed at the onset of mitosis (Buck et al., 2004; Szilagyi et al., 2012). CDK8 has also been implicated in G1 cell cycle commitment in budding yeast, where the mediator complex is recruited to the SBF‐controlled genes by Swi5 transcription factor, before they are acted upon by the Cdc28 (Bhoite et al., 2001; Cosma et al., 2001; Kishi et al., 2008; Szilagyi & Gustafsson, 2013). The implication of CDK8 in the restriction point is further highlighted by its role in maintaining quiescence of vulval precursor cells in *C. elegans*, which start performing superfluous cell divisions after loss of the mediator complex (Clayton et al., 2008; Szilagyi & Gustafsson, 2013).

Despite CDK12/13 being typically classified as tCDKS, similarly to CDK7, they were first identified during cDNA screens for cell‐cycle regulators (Ko et al., 2001; Kohoutek & Blazek, 2012; Marqués et al., 2000). Similarly, the gene encoding their cyclin partner, cyclin K, was first identified through its ability to complement G1 cyclin gene deletion mutants in *S. cerevisiae* (Edwards et al., 1998). The pattern of Cyclin K expression also correlates positively with proliferative capacity (Dai et al., 2012; Lei et al., 2018; Xiang et al., 2014). More recently, the knockdown of either CDK12 or cyclin K in different human cell lines was shown to induce cell cycle arrest in G1 by preventing assembly of the pre‐replicative complex (Lei et al., 2018). In particular, CDK12‐cyclin K complex was found to phosphorylate cyclin E1 in G1 to restrict its ability to interact with CDK2, thereby favoring formation of the pre‐replicative complex. A peak of CDK12 expression in early G1 adds further weight to this discovery (Bertoli et al., 2013; Manavalan et al., 2019). Whether CDK12/cyclin K implication in G1 progression is direct or transcriptional is, however, still under debate. A chemical genetic approach identified CDK12 activity as critical for G1 to S progression in HCT116 cells, but this was shown to depend on its function in activating RNA pol II processivity on key DNA replication genes (Manavalan et al., 2019). Interestingly, depletion of CDK12 in human cells also causes a G2/M arrest, but this may reflect a specific requirement for CDK12 activity for effective transcription of long DNA‐damage response genes (Blazek et al., 2011; Dubbury et al., 2018; Geng et al., 2019; Krajewska et al., 2019; S. Liang et al., 2020; Tellier et al., 2020). CDK12 was also shown to control translation of mitotic regulatory gene mRNAs in human U2OS cells (S. H. Choi et al., 2019). Specifically, CDK12 directly phosphorylates the translation repressor 4E‐BP1, in cooperation with the mTORC1 kinase, to promote its dissociation from the 5′ end of target mRNAs. In comparison, the involvement of CDK13 in the cell cycle is not as well‐understood. However, in gastric cancer cells, which experience increased cell proliferation due to overexpression of the HMGA2 protein, Gene Ontology analysis indicated CDK13 as a cell‐cycle related target of HMGA2 (Z. Wu, Wang, et al., 2021). Rapid cell proliferation in these cells is thought to rely on shortening of the S phase and speeding up progression through the G2/M transition. Joint inhibition of CDK13 and HMGA2 could therefore be antiproliferative (Z. Wu, Wang, et al., 2021). Intriguingly, in breast cancer cells, upregulation of genes involved in S and G2/M progression was caused by inhibiting CDK12/13, further highlighting the role of these kinases in these stages of the cell cycle, and indicating that such effects may be cell type‐dependent (Quereda et al., 2019).

The possible cell‐cycle functions of CDK9 remain elusive. CDK9 levels can oscillate throughout the cell cycle, while cyclin T levels stay relatively constant (Kiernan et al., 2001). However, this is not always the case (Garriga et al., 2003), arguing that CDK9 does not play a major role in cell cycle regulation. In some cell types, such as T cells, signals which induce cell cycle entry can also upregulate cyclin T expression (Garriga et al., 1998; Herrmann et al., 1998), suggesting that it is not the cell cycle but cell activation state that regulates the CDK9‐cyclin T complex (H. Liu & Herrmann, 2005). However, depletion of CDK9 in non‐small cell lung, and head and neck squamous cell carcinoma cell lines induces cell cycle delay with an accumulation of cells in G1 and a corresponding decrease in S phase cells (Cai et al., 2006; Storch & Cordes, 2016). In addition, RNAi‐mediated knockdown of CDK9 in *Drosophila* cells leads to cell cycle arrest in G1 (Anshabo et al., 2021; Yang et al., 2008). Further, CDK9 phosphorylates the Rb protein in vitro and in vivo, which could be linked to the decrease of D‐type cyclins and increase in E‐type cyclins observed after downregulation of this kinase (Graña et al., 1994; Simone et al., 2002; Storch & Cordes, 2016). CDK9 activity was also found necessary for cell cycle recovery after replication stress (D. S. Yu et al., 2010). Recent phosphoproteomic analysis also identified phosphorylation targets of CDK9 that are implicated in the cell cycle, such as FAM122A (PABIR1), which is involved in blocking the G2/M transition, and PCNP, which promotes proliferation (Tellier et al., 2022). Although not as direct as the effects of cell‐cycle CDKs on transcription, tCDKs can therefore influence the cell cycle, to an the extent which is still being uncovered.

## 8 CDKs AND DISEASE

With CDKs implicated in virtually all cellular processes, it is unsurprising that mutation or dysregulation of these kinases can cause a wide range of diseases. Their involvement extends from defects in proliferation, through ischaemia, to rare congenital disorders (Colas, 2020; Łukasik et al., 2021). For example, CDK4 is thought to play a role in the development of some neurodegenerative diseases (Greene et al., 2007; Icreverzi et al., 2015; Łukasik et al., 2021; Mcshea et al., 1997; Sanphui et al., 2013), while CDK5 is specifically implicated in Alzheimer disease, cardiovascular disorders, and diabetes (Arif, 2012; Cicenas & Valius, 2011; Łukasik et al., 2021; Malhotra et al., 2021). As noted above, CDKs can also be co‐opted to help with the replication of viral genomes (Yan et al., 2022). Accordingly, a range of specific and potent CDK inhibitors have been developed, both for clinical and research purposes. Several dozen small‐molecule CDK inhibitors are now being used or tested as treatments for a range of diseases (Abdelmalak et al., 2022; Cicenas & Valius, 2011; Goel et al., 2020; Jhaveri et al., 2021; Marak et al., 2020; Mughal et al., 2023; Roskoski, 2019; Sánchez‐Martínez et al., 2019; Zhang et al., 2021).

### 8.1 Cell‐cycle CDKs contribute to tumorigenesis

The unscheduled cell proliferation that characterizes tumorigenesis is in many cases attributable to decreased requirement for authentic mitogen signaling and so loss of stringent R point control. The first indications that dysregulation of interphase CDK‐cyclin complexes might contribute to this aspect of tumor development came from observations of chromosomal translocations involving the *CCND1* gene, which encodes cyclin D1, in a human parathyroid carcinoma and, most notably, B cell lymphomas (Hsi et al., 1996; Lesage et al., 2005; H. Liu, Wang, & Epner, 2004; Shane, 2001; Thomázy et al., 2002; Vasef et al., 1999; Zhao et al., 2014). A causative role for the resulting cyclin D1 overexpression in tumorigenesis was supported by findings from mouse models in which tissue‐specific promoter‐driven transgenic expression of cyclin D1 led to a high frequency of tumor formation in the corresponding tissue (Fantl et al., 1995; Sicinski et al., 1995). Furthermore, tumorigenesis driven by oncogenes such as Erb‐B2 in transgenic mice was blocked by the simultaneous genetic knock‐out of CDK4 or cyclin D1, or by expression of a mis‐sense mutant form of cyclin D1 that prevents activation of the bound CDK (Landis et al., 2006; Q. Yu et al., 2001, 2006). In familial human melanoma the predisposing alleles include loss‐of‐function mutations in *CDKN2A*, which encodes the p16 CDK inhibitor, and mis‐sense mutations in *CDK4* which result in failure of p16 inhibition of the mutant kinase (Zuo et al., 1996). Thus, cyclin D1, normally expressed in a stringently mitogen‐dependent manner, can be activated pathologically as a result of oncogenic mutations in components of mitogen signaling pathways, and constitutive CDK4/6‐cyclin D1 activity can drive constitutive cell‐cycle commitment as tumors develop. The most widely‐mutated genes across all tumor types include *CDKN2A* and *RB1*, most frequently loss‐of‐function, and *CCND1*, which usually increases in copy number (Aaltonen et al., 2020). As cyclin D1 ablation attenuates tumor growth and activation of cellular senescence, therapeutic targeting of cyclin D1‐associated CDK activity may be therapeutically useful in human cancers (Y. J. Choi et al., 2012).

The process of developing successful CDK inhibitors has been challenging, and many have not progressed beyond early‐stage clinical trials (Asghar et al., 2015; Chohan et al., 2018) largely because they act as competitive inhibitors of ATP binding to CDKs, which are just one sub‐group of some 518 protein kinases in the human proteome. The multiplicity of protein kinases and the limited availability of active, pure preparations of these enzymes have meant that the specificity of even those CDK inhibitors that have been approved for use in the clinic is unclear (see, e.g., Fry et al., 2004). These small‐molecule inhibitors may therefore have biologically significant impacts on multiple protein kinases, and perhaps other ATP‐dependent processes, in patients. There is therefore an urgent need for allosteric CDK inhibitors with increased specificity (Marak et al., 2020; Sánchez‐Martínez et al., 2019).

Despite these caveats, the ATP‐competitive inhibitors palbociclib, ribociclib, and abemaciclib show some specificity for CDK4/CDK6 inhibition in vitro and inhibit the proliferation of cancer cell lines, inducing G1 arrest in an Rb‐dependent manner (Finn et al., 2009). These inhibitors were approved by the U.S. Food and Drug Administration as a therapy against hormone receptor‐positive advanced breast cancer, in conjunction with endocrine agents (Finn et al., 2016; Sánchez‐Martínez et al., 2019). Ribociclib and abemaciclib confer measurable overall survival benefits (Im et al., 2019; Sledge et al., 2020) and they are now being tested in combination with other drugs, immuno‐ and chemotherapy, as well as against other types of cancer (Bonelli et al., 2019; Lynce et al., 2018; Spring et al., 2019). However, evidence is lacking that the clinical responses seen were due to inhibition of CDK4/6 in patients' tumors.

Although CDK2 is not required for proliferation of brain or connective tissue, its inhibition in glioblastoma and osteosarcoma cell lines causes decrease in proliferation of transformed cells (Malumbres & Barbacid, 2009). In this context, it is notable that amplification of the gene encoding the CDK2 partner cyclin E1, is even more common than *CCND1* amplification across numerous human tumor types (Aaltonen et al., 2020). Overexpressed cyclin E1 may be redundant with cyclin D1 in driving R point transit and may contribute to genomic instability by promoting re‐replication of chromosomal DNA.

In line with its role in ensuring developmental viability, and its nonredundant role in the cell cycle, CDK1 is one of the least mutated CDKs in cancer (Asghar et al., 2015; Otto & Sicinski, 2017; Peyressatre et al., 2015). In tumors, CDK1 activity is often dysregulated through indirect mutations in DNA damage response pathways, or loss of CDK inhibitors (Asghar et al., 2015). However, there is growing evidence that CDK1 can act as a driver of cancer development and progression (Otto & Sicinski, 2017; Sofi et al., 2022). Overexpression of CDK1 has been found to be a biomarker in a number of different types of cancer, including lung, pancreas and sarcomas (M. Li et al., 2020; Q. Li, Zhang, et al., 2019; Piao et al., 2019; Yamamura et al., 2020). Similarly, overexpression of its partner cyclin B is often associated with poor prognosis in, for instance, breast cancer patients (Agarwal et al., 2010; Winters et al., 2001). Inhibition of CDK1 can induce apoptosis in several types of malignancy (Ongkeko et al., 1995; Otto & Sicinski, 2017) and could prove effective against cancer stem cells, particularly in gliomas and pancreatic cancers (Sánchez‐Martínez et al., 2019). In the absence of evidence to the contrary, it is even possible that clinical responses to CDK inhibition are due, in part at least, to off‐target inhibition of CDK1.

### 8.2 tCDKs are dysregulated in cancer

As malignant cells generally rely on a higher transcriptional output than healthy cells, often relying on oncogenic transcription factors or super‐enhancers, it is not surprising that dysregulation of key tCDKs is implicated in a range of cancers. As a result, tCDKs are currently seen as important pharmaceutical targets and biomarkers of cancer, with several tCDK inhibitors currently in clinical trials for cancer treatment (Franco & Kraus, 2015; Galbraith et al., 2019; Parua & Fisher, 2020; Sánchez‐Martínez et al., 2019).

The dual actions of the highly related kinases CDK8/CDK19 (Table 4) have led them to be implicated in cancer development as both drivers or suppressors of tumorigenesis. Inhibition of both kinases, whose catalytic domains share 94% amino acid sequence identity, by cortistatin A leads to decreased proliferation of malignant cells (Chou et al., 2020; Pelish et al., 2015). In addition, overexpression of CDK8 can contribute to the development of melanoma, as well as colorectal, gastric and breast cancers (Chohan et al., 2018; Peyressatre et al., 2015). In prostate cancer cells, inhibition of CDK8/19 leads to premature G1/S transition and cell death (Nakamura et al., 2018). However, CDK8 can also inhibit tumor growth, for instance in the case of endometrial cancer. It was shown that ectopic expression of CDK8 inhibited proliferation in the KLE cancer cell line, and that it could even block growth of a mouse tumor model in vivo (Gu et al., 2013). In addition to cortistatin A, a number of CDK8/CDK19 inhibitors are currently under development but none has yet reached the clinic (Galbraith et al., 2019).

Interest in designing a specific CDK9 inhibitor began when it was discovered that pan‐CDK inhibitors, for instance flavopiridol, potently inhibit this kinase. CDK9 is hyperactivated in a number of cancers, particularly hematological malignancies (Chohan et al., 2018; Galbraith et al., 2019). The involvement of CDK9 in lymphoma development is also connected to its role in maintaining the expression of short‐lived anti‐apoptotic mRNAs, such as *MCL1*. It is further recruited to chromatin by various cancer‐dependent transcription factors and super‐enhancers (Galbraith et al., 2019). Because of its central role in transcription elongation, CDK9 remains a high‐priority target, with a number of inhibitors currently undergoing clinical trials (Galbraith et al., 2019).

Like CDK8/CDK19, CDK12, and CDK13 have been shown to have both tumor‐permissive and suppressive functions. CDK12 is recognized as a significant player in genomic instability of cancers and its loss‐of‐function mutations have been implicated in advanced ovarian and breast malignancies (Cheng et al., 2022; Chohan et al., 2018; Galbraith et al., 2019; H. Liu et al., 2021). On the other hand, its overexpression is associated with HER2‐positive breast cancers and increased levels of ERBB2 (Quereda et al., 2019). Importantly, depletion of CDK12 preferentially decreases the expression of DNA damage response (DDR) genes (Blazek et al., 2011; Manavalan et al., 2019; S. H. Choi et al., 2020; Dubbury et al., 2018; Krajewska et al., 2019). This allows for sensitization of the tumor to synthetic‐lethal therapy, for instance with poly‐ADP ribose polymerase (PARP) inhibitors (Chou et al., 2020; Parua & Fisher, 2020). Despite being less studied, CDK13 has also been found to be amplified in some primary liver and colon cancers (Galbraith et al., 2019). Although a clinical inhibitor of CDK12 or CDK13 is still in the works, there currently exists a range of research‐grade tools used to inhibit these kinases. Further, as seen with the example of PARP inhibitors, CDK12/CDK13 inhibitors can synergize with other therapeutic agents (Galbraith et al., 2019; Niu et al., 2022; Quereda et al., 2019; Tadesse et al., 2020). Preliminary data suggest triple‐negative breast cancer as a potential therapeutic target for CDK12/13 inhibition, which would depend on suppressing the DNA damage response pathway and leading to sensitization of malignant cells to apoptosis (Hopkins & Zou, 2019; Quereda et al., 2019; Tadesse et al., 2020).

Perhaps the biggest success in therapeutic targeting of tCDKs is seen in the example of CDK7, with multiple selective inhibitors currently in Phase I/II clinical trials (Kovalová et al., 2023; Sava et al., 2020). The dual actions of CDK7, which participates in both regulation of transcription and the cell cycle, have made it an important player in tumorigenesis. Its overactivation is associated with gastric, breast, ovarian, and liver cancers, and it has been suggested that the malignant cells show greater dependency on elevated CDK7 levels than healthy cells (Galbraith et al., 2019; Sava et al., 2020; Wang et al., 2015). THZ1 is a covalent CDK inhibitor, and although it primarily targets CDK7, it also has some anti‐CDK12/CDK13 activity (Sánchez‐Martínez et al., 2019). Encouragingly, treating cells advanced breast cancer cells with THZ1 leads to apoptotic cell death (Franco & Kraus, 2015; Wang et al., 2015). Several other groups also showed that selective inhibition of CDK7 leads to cell cycle arrest, apoptosis, and decreased transcription levels, especially of genes associated with super‐enhancers (Galbraith et al., 2019; Sava et al., 2020). Importantly, inhibition of CDK7 shows promise in treating cancers with heightened transcription, even without the presence of any apparent oncogenic driver mutations (Wang et al., 2015). Additionally, CDK7 inhibitors show promise of synergistic treatment, for example with pro‐apoptotic agents (He et al., 2022; Kalan et al., 2017). In the same manner, colorectal cancer cells treated with the antimetabolite 5‐fluorouracil became sensitized to reversible or covalent CDK7 inhibitors, and showed increased rates of apoptotic cell death (Kalan et al., 2017).

## 9 CONCLUSION

The picture emerging from the field of CDK biology is one of great complexity. The multitasking CDKs can be seen as a versatile group of kinases, which help to receive, interpret, and act on a variety of endogenous and exogenous signals. The redundancy between individual family members and low selectivity of inhibitors has made investigation of CDK roles challenging. Examining the direct, primary actions of these kinases was further complicated by the use of long‐term inhibition approaches, such as siRNA‐mediated knockdown. Despite this, their molecular functions are now coming more sharply into focus. In cancer, tumors acquire dependencies on processes guarded by the CDKs, which may become rate‐limiting for tumor growth and progression. As the involvement of CDK deregulation in diseases becomes clearer, more therapeutic opportunities arise.

It is also increasingly apparent that a binary distinction between cell‐cycle and transcriptional CDKs is inappropriate. Rather, the involvement of several CDKs in both aspects of biology suggests an evolutionarily ancient role in the co‐ordination of cell proliferation and gene expression.

## AUTHOR CONTRIBUTIONS

**Aleksandra J. Pluta:** Conceptualization (lead); writing – original draft (lead); writing – review and editing (lead). **Cécilia Studniarek:** Writing – original draft (supporting); writing – review and editing (supporting). **Shona Murphy:** Conceptualization (equal); funding acquisition (lead); supervision (equal); writing – review and editing (lead). **Chris J. Norbury:** Conceptualization (equal); supervision (equal); writing – review and editing (equal).

## FUNDING INFORMATION

This work was supported by Wellcome Trust Investigator Grant 210641/Z/18/Z to SM.

## CONFLICT OF INTEREST STATEMENT

The authors declare no conflicts of interest.

## RELATED WIREs ARTICLES

Transcription and splicing: A two‐way street

## Data Availability

Data sharing is not applicable to this article as no new data were created or analyzed in this study.
